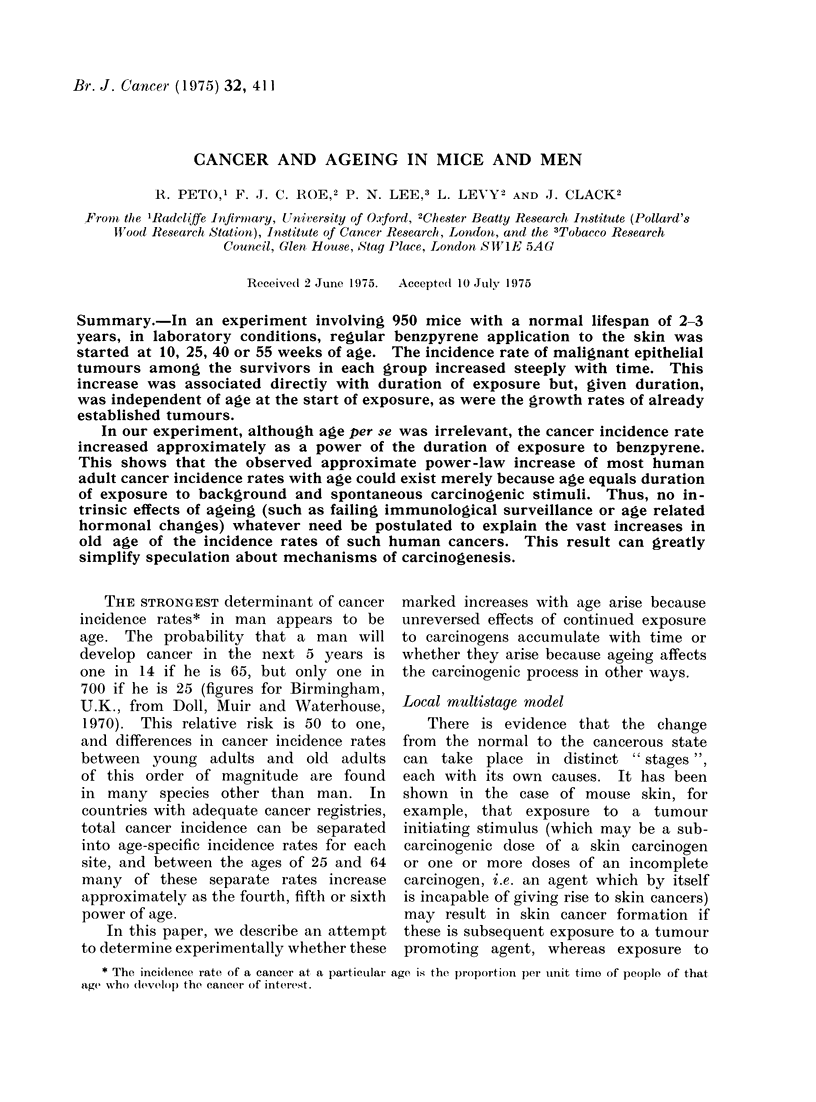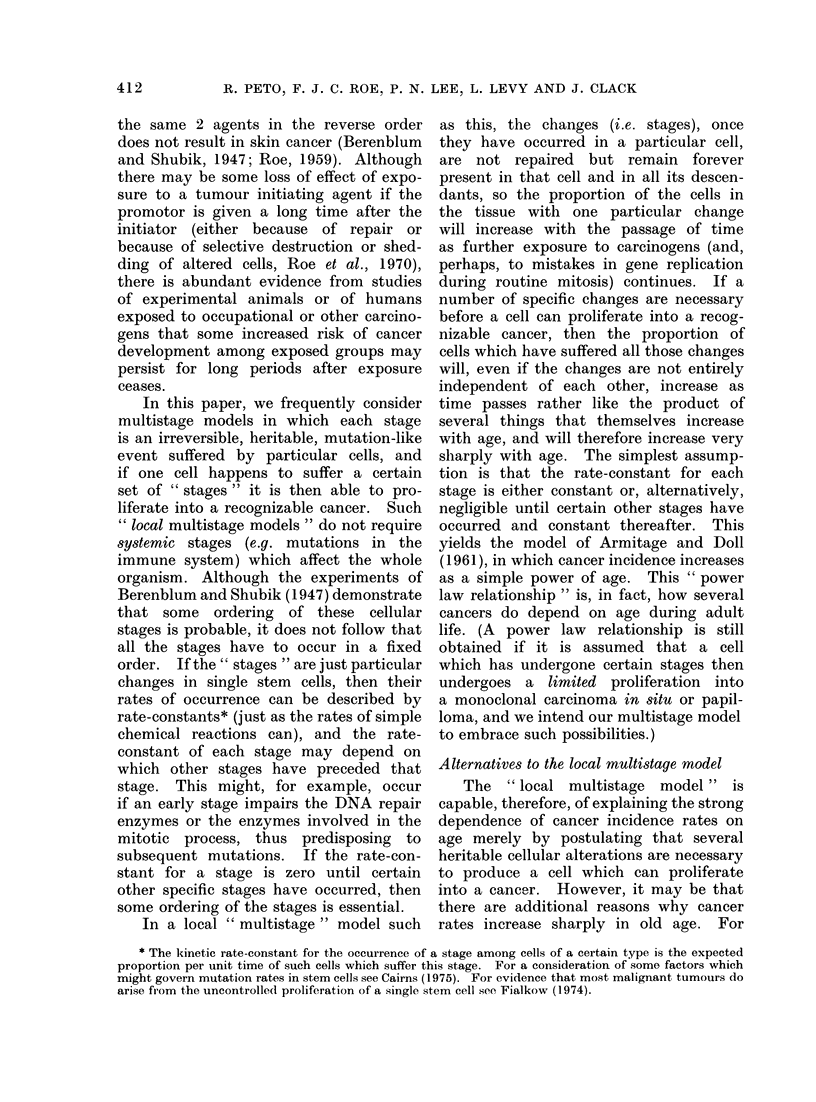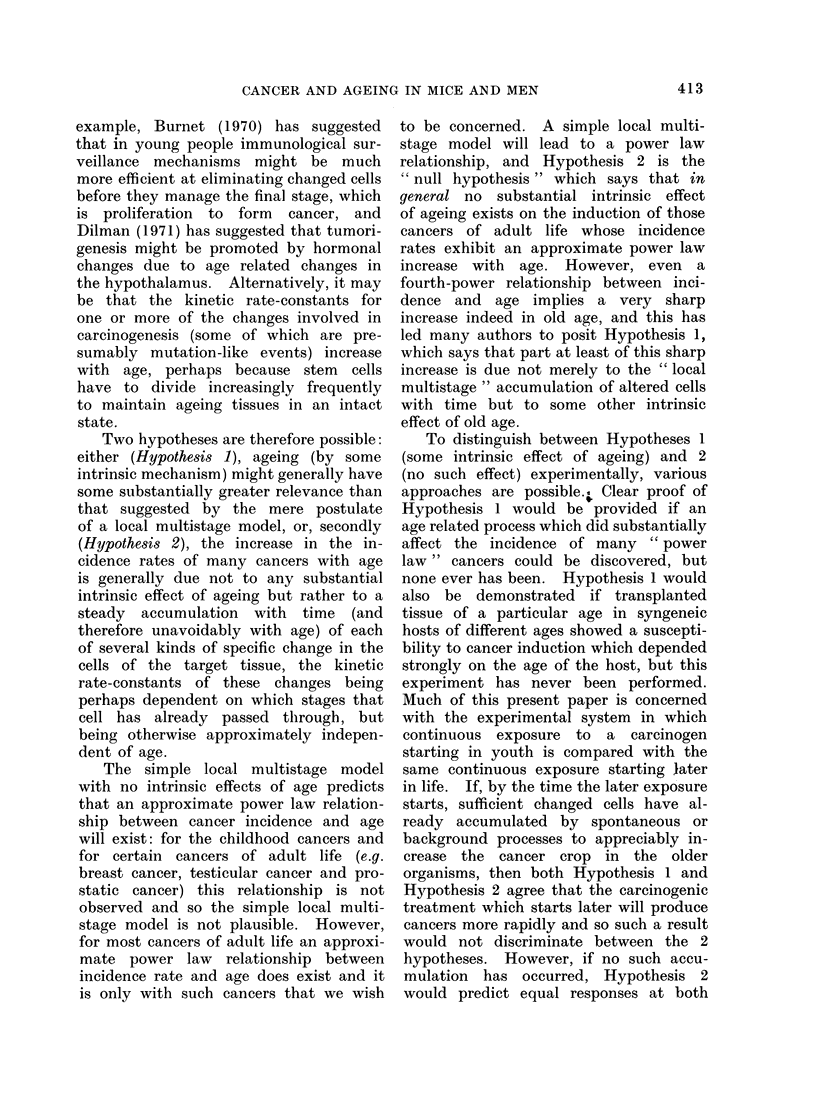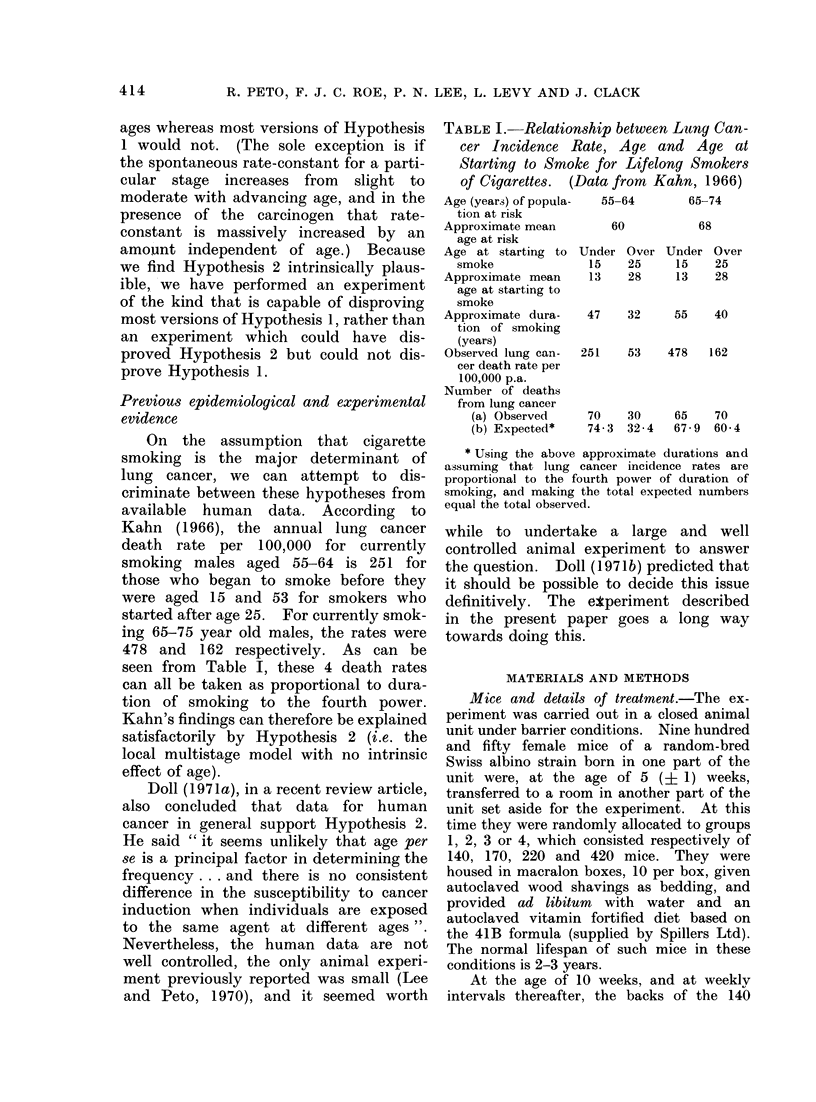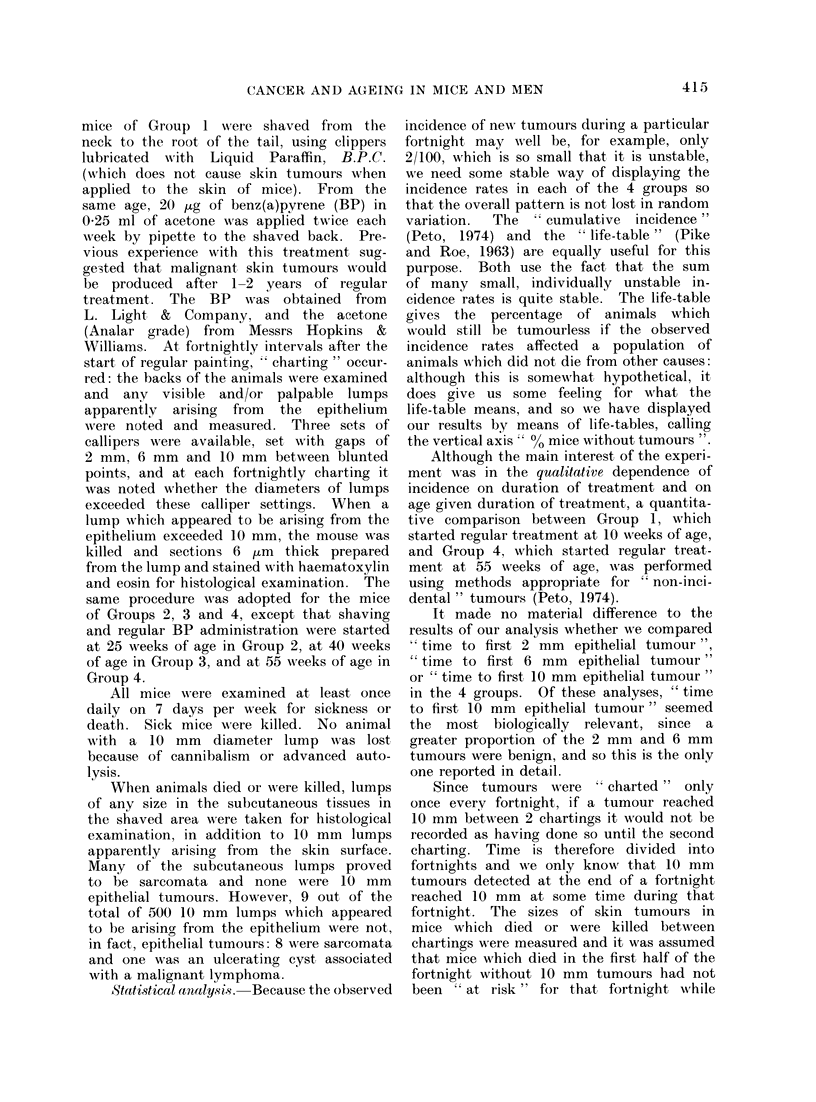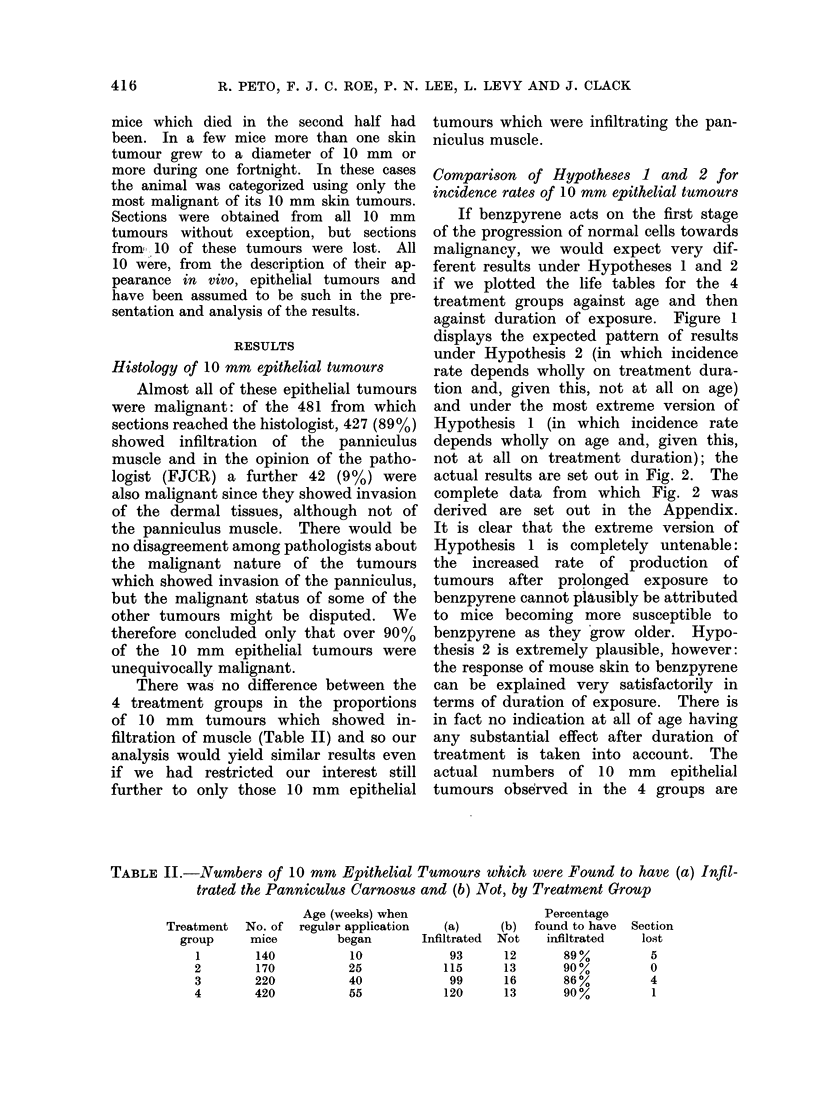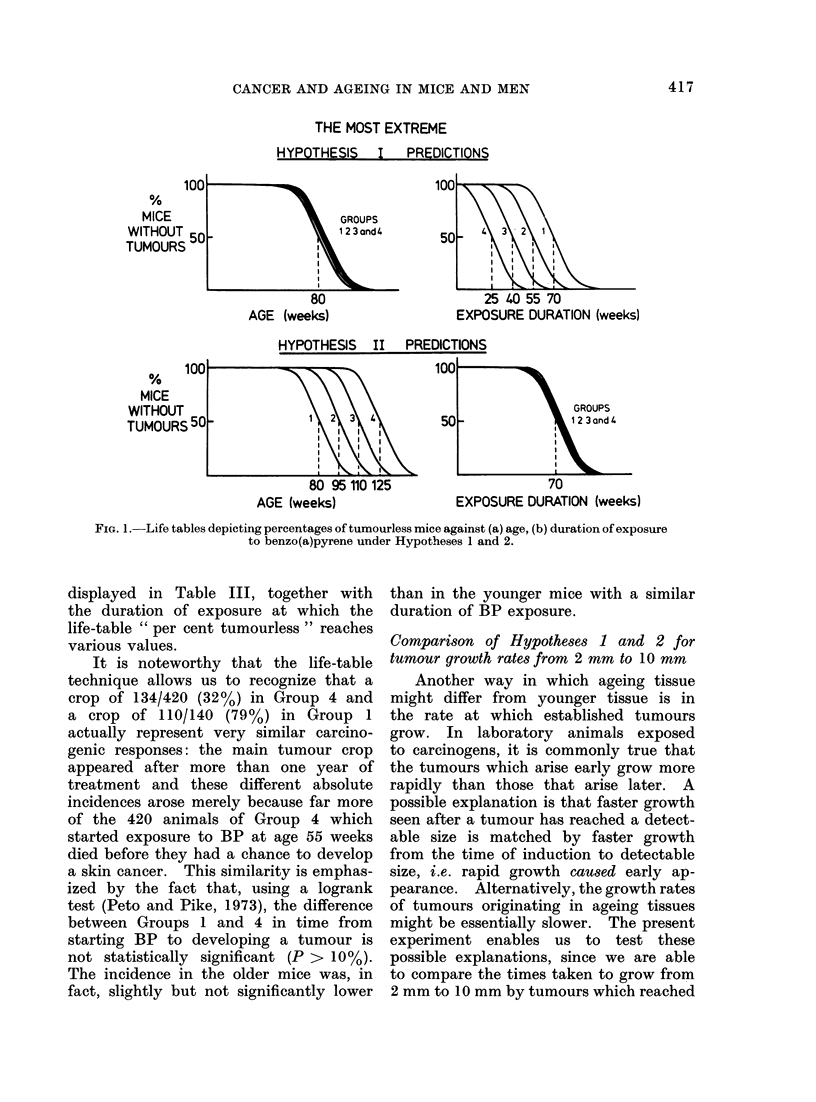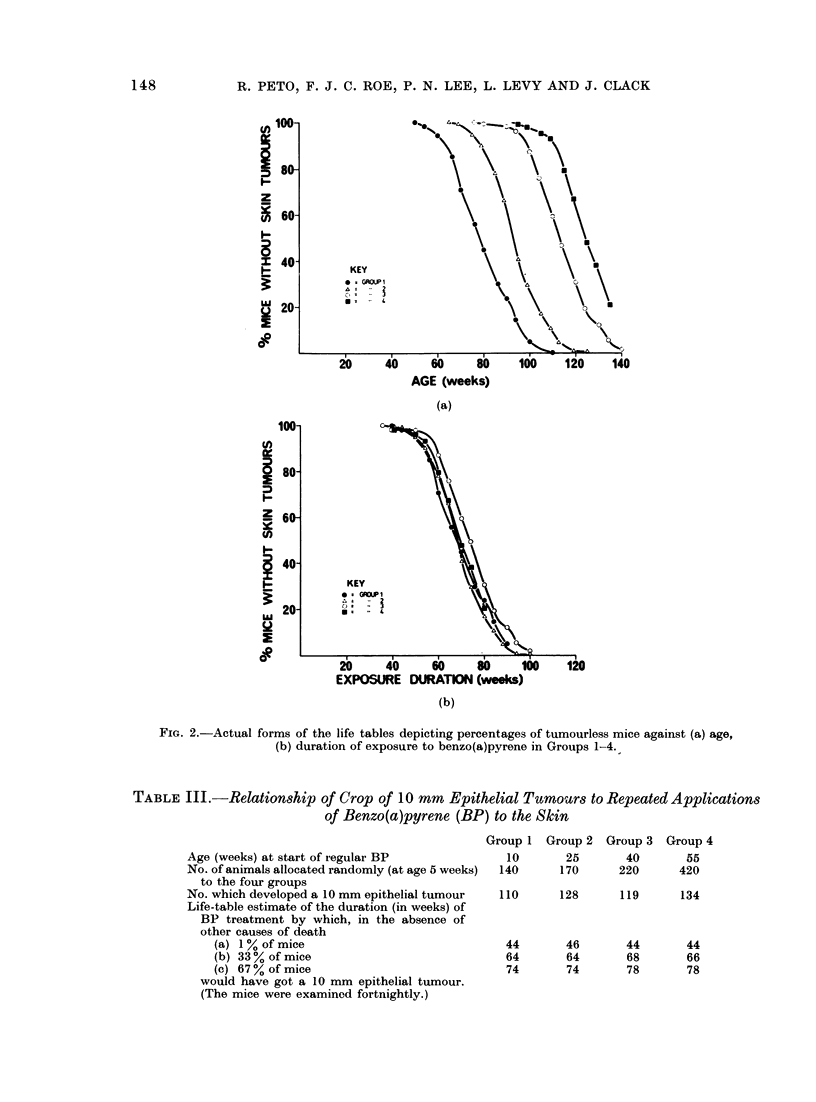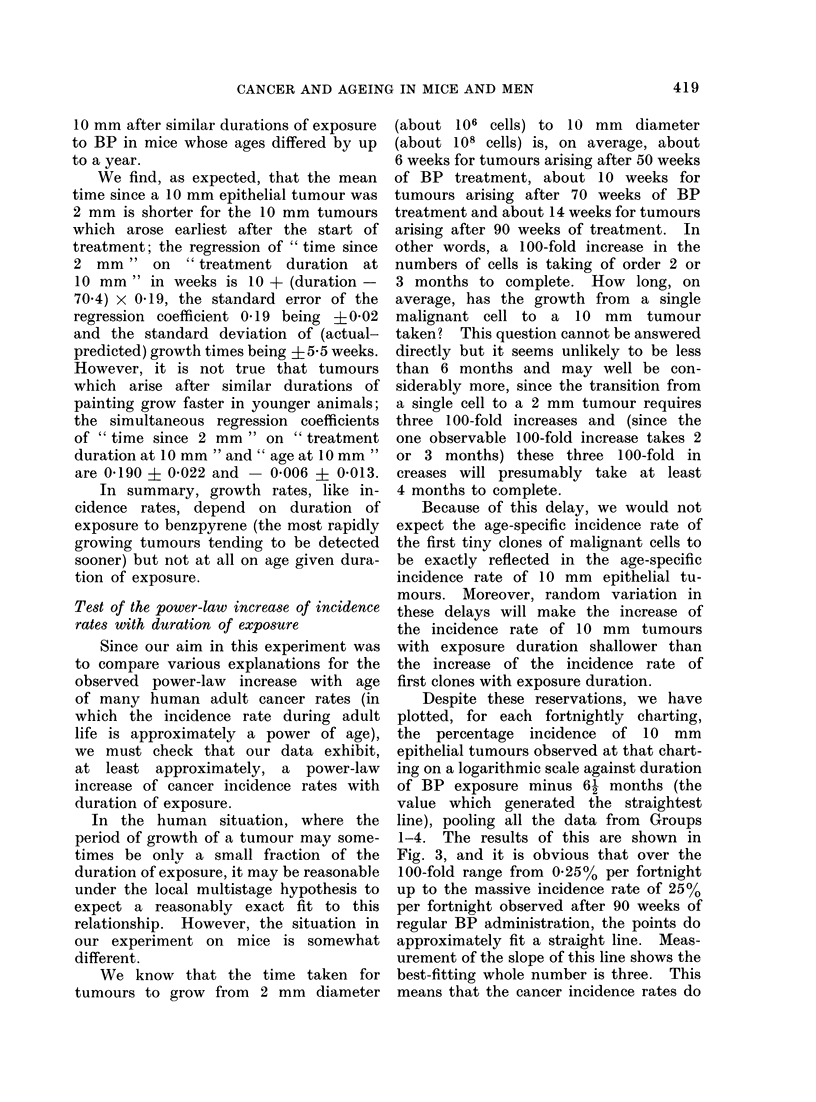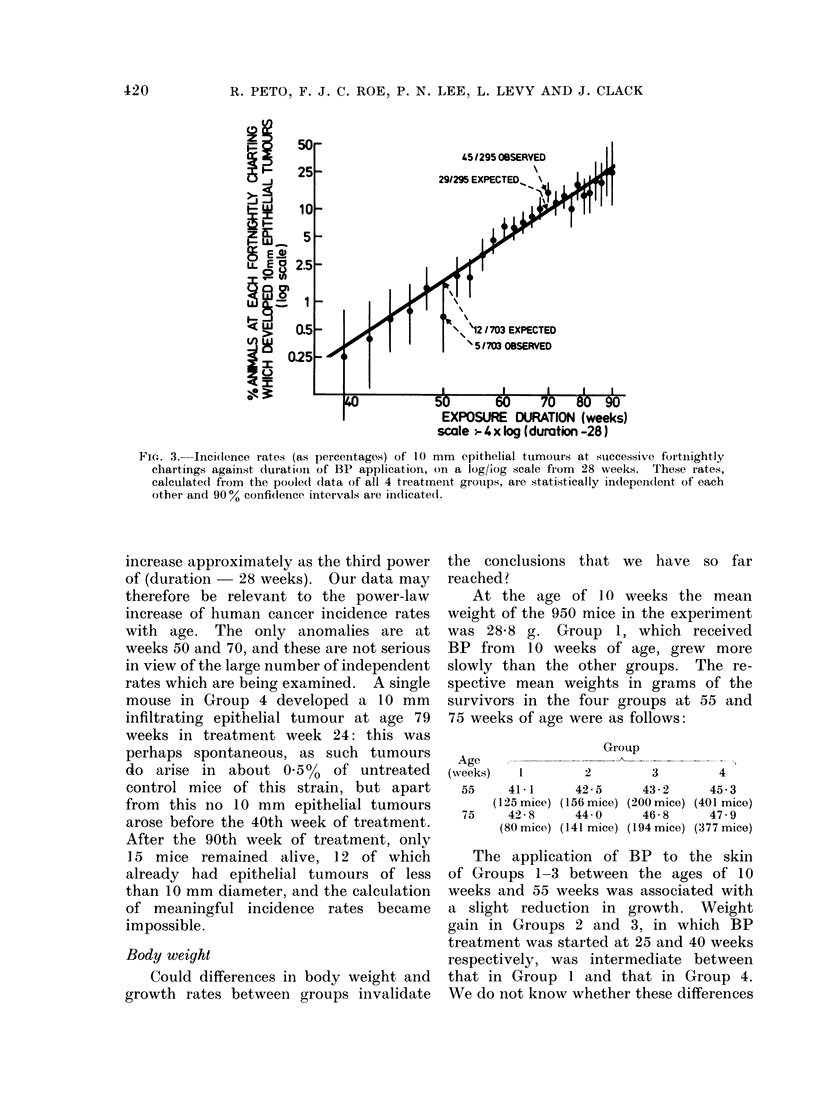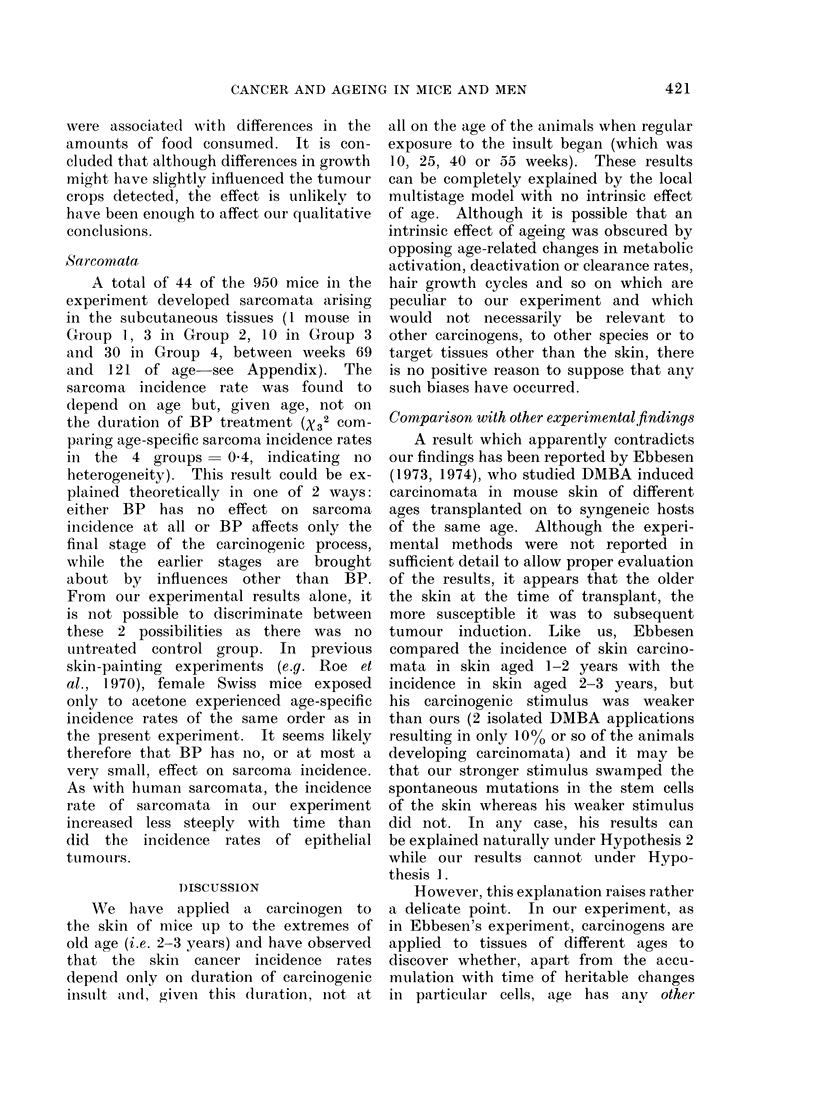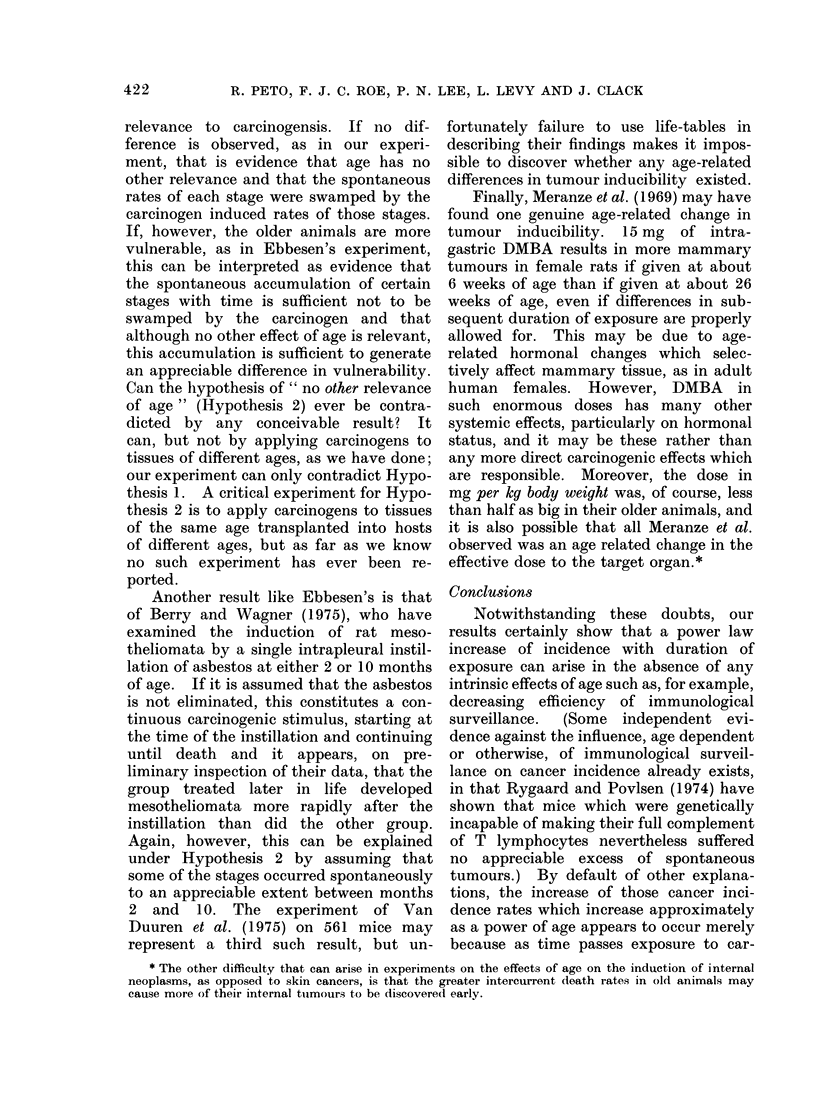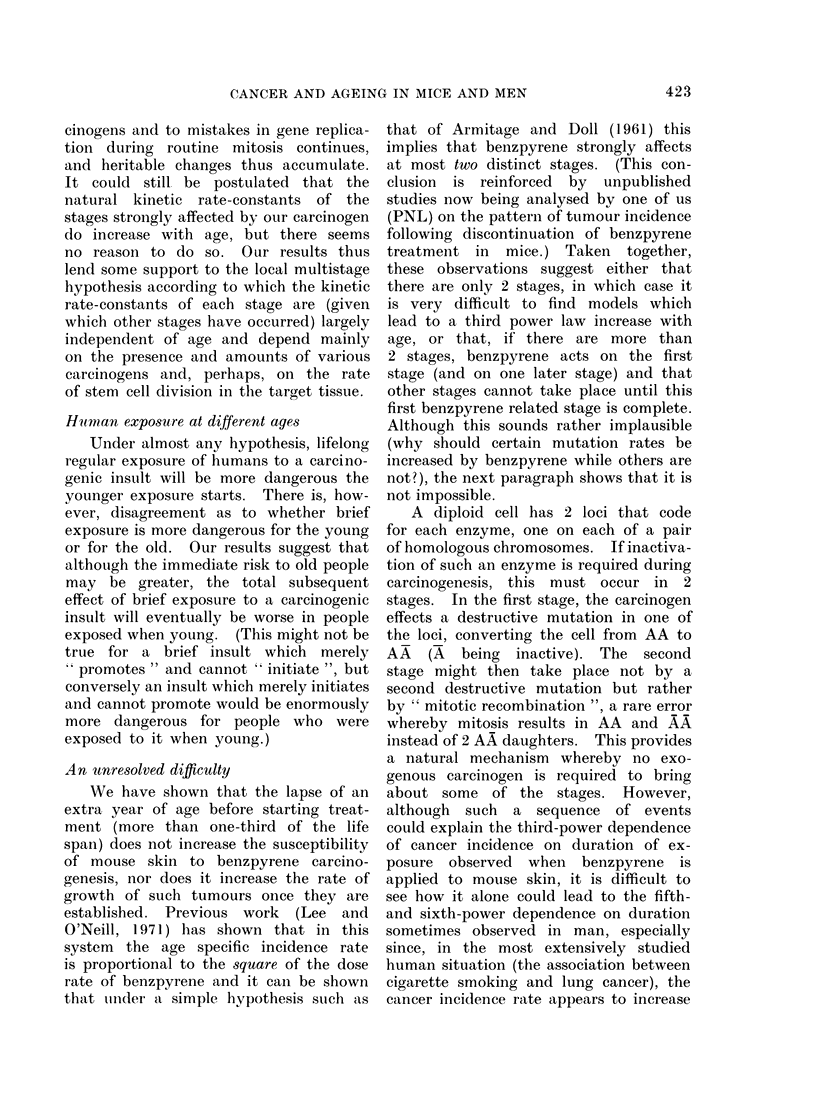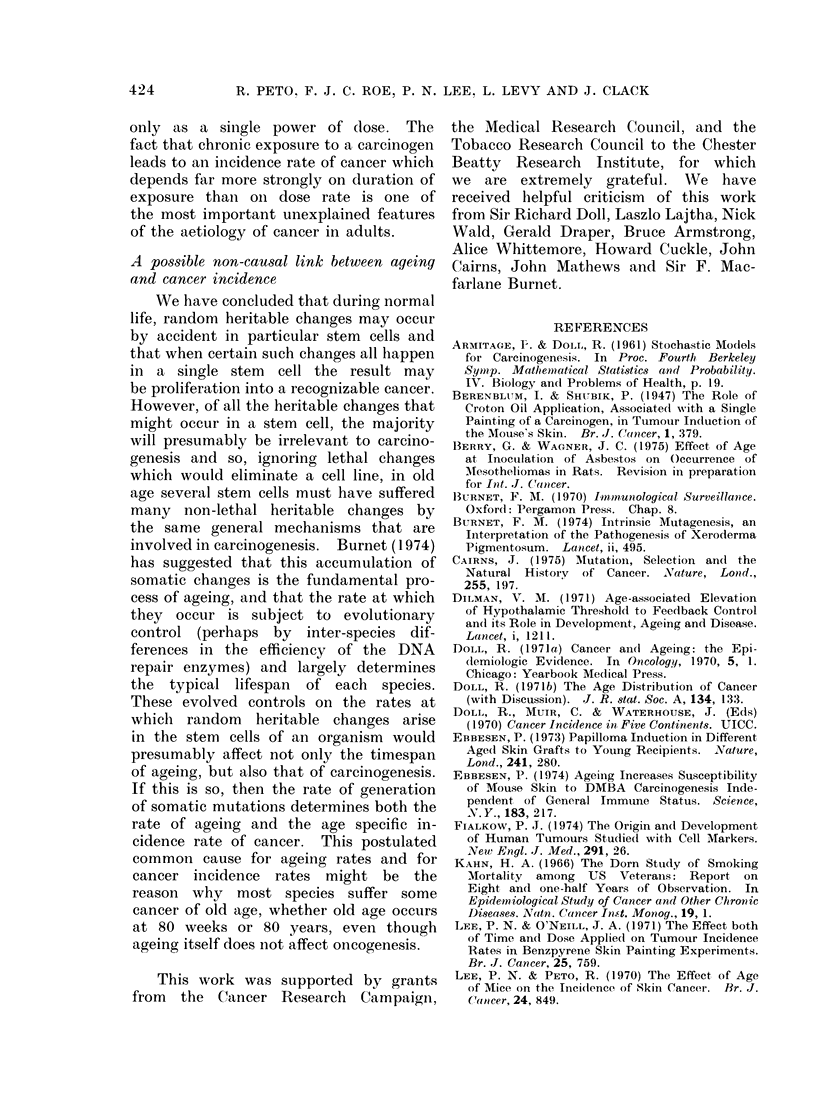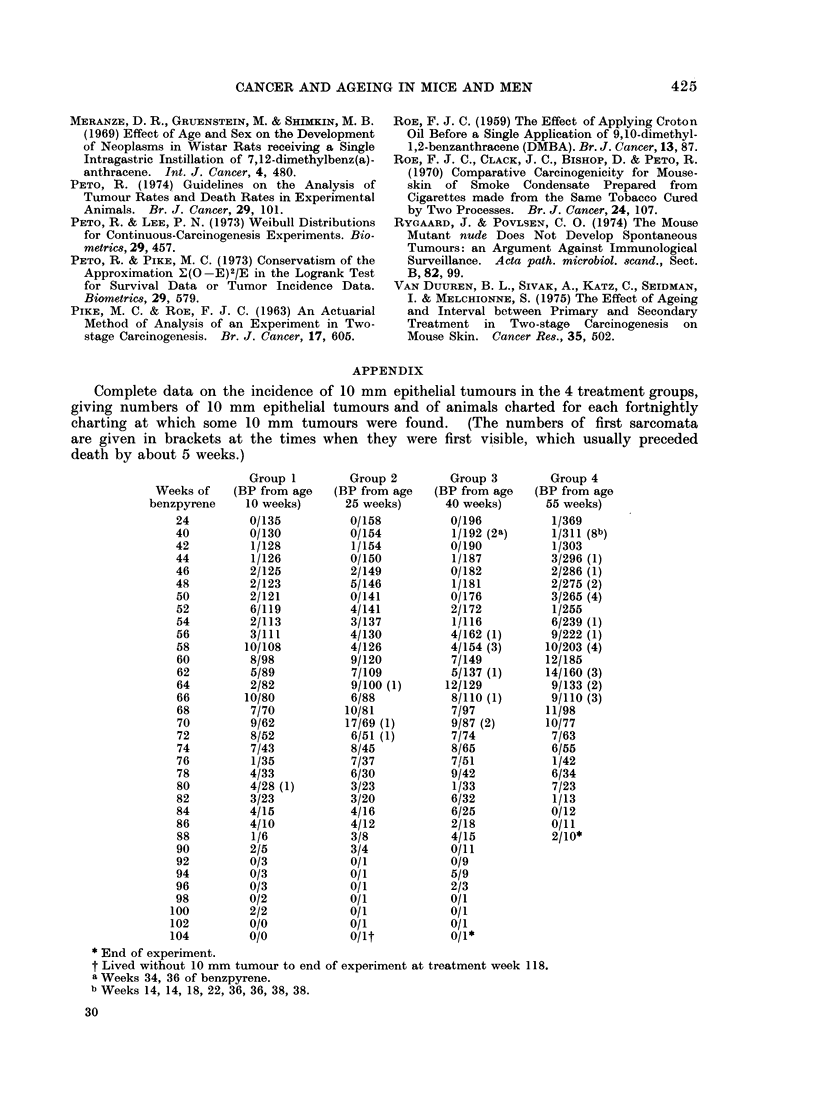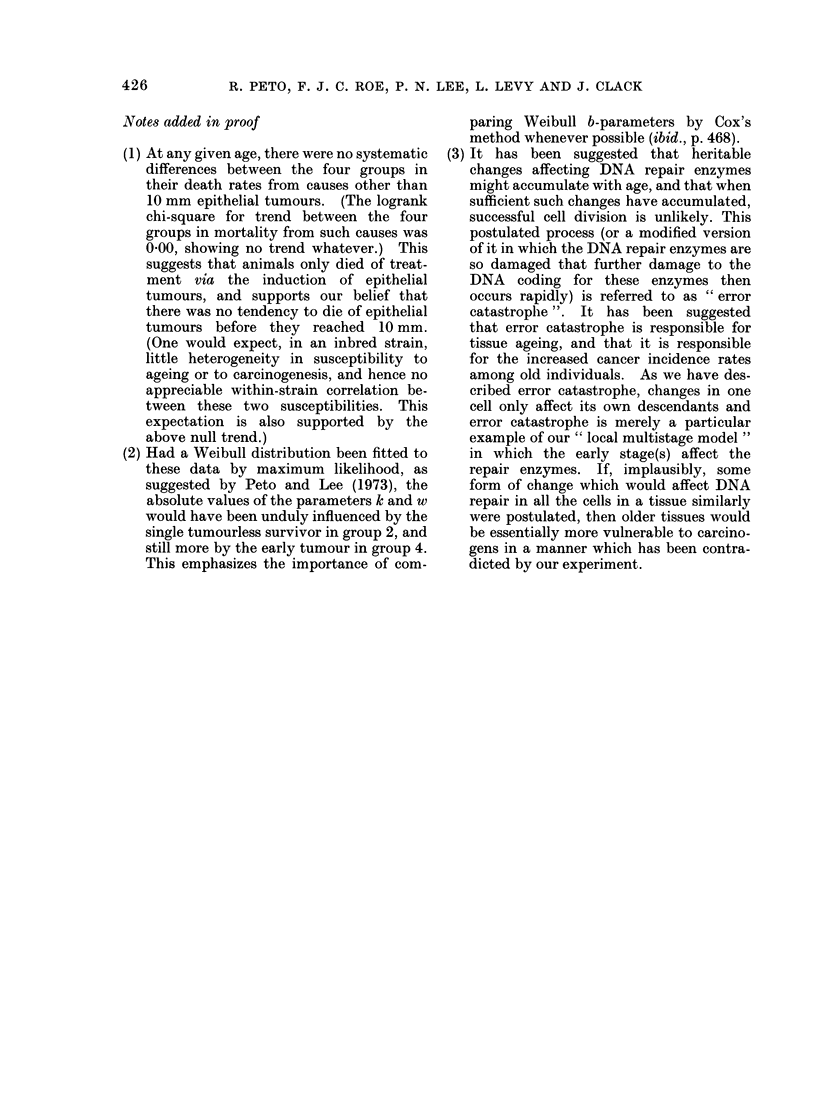# Cancer and ageing in mice and men.

**DOI:** 10.1038/bjc.1975.242

**Published:** 1975-10

**Authors:** R. Peto, F. J. Roe, P. N. Lee, L. Levy, J. Clack

## Abstract

In an experiment involving 950 mice with a normal lifespan of 2-3 years, in laboratory conditions, regular benzpyrene application to the skin was started at 10, 25, 40 or 55 weeks of age. The incidence rate of malignant epithelial tumours among the survivors in each group increased steeply with time. This increase was associated directly with duration of exposure but, given duration, was independent of age at the start of exposure, as were the growth rates of already established tumours. In our experiment, although age per se was irrelevant, the cancer incidence rate increased approximately as a power of the duration of exposure to benzpyrene. This shows that the observed approximate power-law increase of most human adult cancer incidence rates with age could exist merely because age equals duration of exposure to background and spontaneous carcinogenic stimuli. Thus, no intrinsic effects of ageing (such as failing immunological surveillance or age related hormonal changes) whatever need to postulated to explain the vast increases in old age of the incidence rates of such human cancers. This result can greatly simplify speculation about mechanisms of carcinogenesis.


*Br. J. Cancer* (1975) **32,** 411

### CANCER AND AGEING IN MICE AND MEN

R. PETO,[1] F. J. C. ROE,[2] P. N. LEE,[3] L. LEVY[2] AND J. CLACK[2]

*From the [1]Radcliffe Infirmary, University of Oxford, [2]Chester Beatty Research Institute (Pollard's Wood Research Station), Institute of Cancer Research, London, and the [3]Tobacco Research Council, Glen House, Stag Place, London SW1E 5AG*

Received 2 June 1975. Accepted 10 July 1975

**Summary.—In an experiment involving 950 mice with a normal lifespan of 2–3 years, in laboratory conditions, regular benzpyrene application to the skin was started at 10, 25, 40 or 55 weeks of age. The incidence rate of malignant epithelial tumours among the survivors in each group increased steeply with time. This increase was associated directly with duration of exposure but, given duration, was independent of age at the start of exposure, as were the growth rates of already established tumours.**

**In our experiment, although age *per se* was irrelevant, the cancer incidence rate increased approximately as a power of the duration of exposure to benzpyrene. This shows that the observed approximate power-law increase of most human adult cancer incidence rates with age could exist merely because age equals duration of exposure to background and spontaneous carcinogenic stimuli. Thus, no intrinsic effects of ageing (such as failing immunological surveillance or age related hormonal changes) whatever need be postulated to explain the vast increases in old age of the incidence rates of such human cancers. This result can greatly simplify speculation about mechanisms of carcinogenesis.**

THE STRONGEST determinant of cancer incidence rates* in man appears to be age. The probability that a man will develop cancer in the next 5 years is one in 14 if he is 65, but only one in 700 if he is 25 (figures for Birmingham, U.K., from Doll, Muir and Waterhouse, 1970). This relative risk is 50 to one, and differences in cancer incidence rates between young adults and old adults of this order of magnitude are found in many species other than man. In countries with adequate cancer registries, total cancer incidence can be separated into age-specific incidence rates for each site, and between the ages of 25 and 64 many of these separate rates increase approximately as the fourth, fifth or sixth power of age.

In this paper, we describe an attempt to determine experimentally whether these marked increases with age arise because unreversed effects of continued exposure to carcinogens accumulate with time or whether they arise because ageing affects the carcinogenic process in other ways.

*Local multistage model*

There is evidence that the change from the normal to the cancerous state can take place in distinct "stages", each with its own causes. It has been shown in the case of mouse skin, for example, that exposure to a tumour initiating stimulus (which may be a sub-carcinogenic dose of a skin carcinogen or one or more doses of an incomplete carcinogen, *i.e.* an agent which by itself is incapable of giving rise to skin cancers) may result in skin cancer formation if these is subsequent exposure to a tumour promoting agent, whereas exposure to

* The incidence rate of a cancer at a particular age is the proportion per unit time of people of that age who develop the cancer of interest.

the same 2 agents in the reverse order does not result in skin cancer (Berenblum and Shubik, 1947; Roe, 1959). Although there may be some loss of effect of exposure to a tumour initiating agent if the promotor is given a long time after the initiator (either because of repair or because of selective destruction or shedding of altered cells, Roe *et al.*, 1970), there is abundant evidence from studies of experimental animals or of humans exposed to occupational or other carcinogens that some increased risk of cancer development among exposed groups may persist for long periods after exposure ceases.

In this paper, we frequently consider multistage models in which each stage is an irreversible, heritable, mutation-like event suffered by particular cells, and if one cell happens to suffer a certain set of " stages " it is then able to proliferate into a recognizable cancer. Such " *local* multistage models " do not require *systemic* stages (*e.g.* mutations in the immune system) which affect the whole organism. Although the experiments of Berenblum and Shubik (1947) demonstrate that some ordering of these cellular stages is probable, it does not follow that all the stages have to occur in a fixed order. If the " stages " are just particular changes in single stem cells, then their rates of occurrence can be described by rate-constants* (just as the rates of simple chemical reactions can), and the rate-constant of each stage may depend on which other stages have preceded that stage. This might, for example, occur if an early stage impairs the DNA repair enzymes or the enzymes involved in the mitotic process, thus predisposing to subsequent mutations. If the rate-constant for a stage is zero until certain other specific stages have occurred, then some ordering of the stages is essential.

In a local " multistage " model such as this, the changes (*i.e.* stages), once they have occurred in a particular cell, are not repaired but remain forever present in that cell and in all its descendants, so the proportion of the cells in the tissue with one particular change will increase with the passage of time as further exposure to carcinogens (and, perhaps, to mistakes in gene replication during routine mitosis) continues. If a number of specific changes are necessary before a cell can proliferate into a recognizable cancer, then the proportion of cells which have suffered all those changes will, even if the changes are not entirely independent of each other, increase as time passes rather like the product of several things that themselves increase with age, and will therefore increase very sharply with age. The simplest assumption is that the rate-constant for each stage is either constant or, alternatively, negligible until certain other stages have occurred and constant thereafter. This yields the model of Armitage and Doll (1961), in which cancer incidence increases as a simple power of age. This " power law relationship " is, in fact, how several cancers do depend on age during adult life. (A power law relationship is still obtained if it is assumed that a cell which has undergone certain stages then undergoes a *limited* proliferation into a monoclonal carcinoma *in situ* or papilloma, and we intend our multistage model to embrace such possibilities.)

### *Alternatives to the local multistage model*

The " local multistage model " is capable, therefore, of explaining the strong dependence of cancer incidence rates on age merely by postulating that several heritable cellular alterations are necessary to produce a cell which can proliferate into a cancer. However, it may be that there are additional reasons why cancer rates increase sharply in old age. For

* The kinetic rate-constant for the occurrence of a stage among cells of a certain type is the expected proportion per unit time of such cells which suffer this stage. For a consideration of some factors which might govern mutation rates in stem cells see Cairns (1975). For evidence that most malignant tumours do arise from the uncontrolled proliferation of a single stem cell see Fialkow (1974).

example, Burnet (1970) has suggested that in young people immunological surveillance mechanisms might be much more efficient at eliminating changed cells before they manage the final stage, which is proliferation to form cancer, and Dilman (1971) has suggested that tumorigenesis might be promoted by hormonal changes due to age related changes in the hypothalamus. Alternatively, it may be that the kinetic rate-constants for one or more of the changes involved in carcinogenesis (some of which are presumably mutation-like events) increase with age, perhaps because stem cells have to divide increasingly frequently to maintain ageing tissues in an intact state.

Two hypotheses are therefore possible: either (*Hypothesis 1*), ageing (by some intrinsic mechanism) might generally have some substantially greater relevance than that suggested by the mere postulate of a local multistage model, or, secondly (*Hypothesis 2*), the increase in the incidence rates of many cancers with age is generally due not to any substantial intrinsic effect of ageing but rather to a steady accumulation with time (and therefore unavoidably with age) of each of several kinds of specific change in the cells of the target tissue, the kinetic rate-constants of these changes being perhaps dependent on which stages that cell has already passed through, but being otherwise approximately independent of age.

The simple local multistage model with no intrinsic effects of age predicts that an approximate power law relationship between cancer incidence and age will exist: for the childhood cancers and for certain cancers of adult life (*e.g.* breast cancer, testicular cancer and prostatic cancer) this relationship is not observed and so the simple local multistage model is not plausible. However, for most cancers of adult life an approximate power law relationship between incidence rate and age does exist and it is only with such cancers that we wish to be concerned. A simple local multistage model will lead to a power law relationship, and Hypothesis 2 is the " null hypothesis " which says that *in general* no substantial intrinsic effect of ageing exists on the induction of those cancers of adult life whose incidence rates exhibit an approximate power law increase with age. However, even a fourth-power relationship between incidence and age implies a very sharp increase indeed in old age, and this has led many authors to posit Hypothesis 1, which says that part at least of this sharp increase is due not merely to the " local multistage " accumulation of altered cells with time but to some other intrinsic effect of old age.

To distinguish between Hypotheses 1 (some intrinsic effect of ageing) and 2 (no such effect) experimentally, various approaches are possible. Clear proof of Hypothesis 1 would be provided if an age related process which did substantially affect the incidence of many " power law " cancers could be discovered, but none ever has been. Hypothesis 1 would also be demonstrated if transplanted tissue of a particular age in syngeneic hosts of different ages showed a susceptibility to cancer induction which depended strongly on the age of the host, but this experiment has never been performed. Much of this present paper is concerned with the experimental system in which continuous exposure to a carcinogen starting in youth is compared with the same continuous exposure starting later in life. If, by the time the later exposure starts, sufficient changed cells have already accumulated by spontaneous or background processes to appreciably increase the cancer crop in the older organisms, then both Hypothesis 1 and Hypothesis 2 agree that the carcinogenic treatment which starts later will produce cancers more rapidly and so such a result would not discriminate between the 2 hypotheses. However, if no such accumulation has occurred, Hypothesis 2 would predict equal responses at both ages whereas most versions of Hypothesis 1 would not. (The sole exception is if the spontaneous rate-constant for a particular stage increases from slight to moderate with advancing age, and in the presence of the carcinogen that rate-constant is massively increased by an amount independent of age.) Because we find Hypothesis 2 intrinsically plausible, we have performed an experiment of the kind that is capable of disproving most versions of Hypothesis 1, rather than an experiment which could have disproved Hypothesis 2 but could not disprove Hypothesis 1.

*Previous epidemiological and experimental evidence*

On the assumption that cigarette smoking is the major determinant of lung cancer, we can attempt to discriminate between these hypotheses from available human data. According to Kahn (1966), the annual lung cancer death rate per 100,000 for currently smoking males aged 55–64 is 251 for those who began to smoke before they were aged 15 and 53 for smokers who started after age 25. For currently smoking 65–75 year old males, the rates were 478 and 162 respectively. As can be seen from Table I, these 4 death rates can all be taken as proportional to duration of smoking to the fourth power. Kahn's findings can therefore be explained satisfactorily by Hypothesis 2 (*i.e.* the local multistage model with no intrinsic effect of age).

Doll (1971*a*), in a recent review article, also concluded that data for human cancer in general support Hypothesis 2. He said "it seems unlikely that age *per se* is a principal factor in determining the frequency . . . and there is no consistent difference in the susceptibility to cancer induction when individuals are exposed to the same agent at different ages". Nevertheless, the human data are not well controlled, the only animal experiment previously reported was small (Lee and Peto, 1970), and it seemed worth while to undertake a large and well controlled animal experiment to answer the question. Doll (1971*b*) predicted that it should be possible to decide this issue definitively. The experiment described in the present paper goes a long way towards doing this.

TABLE I.—*Relationship between Lung Cancer Incidence Rate, Age and Age at Starting to Smoke for Lifelong Smokers of Cigarettes.* (*Data from Kahn,* 1966)

| | | | | |
|---|---|---|---|---|
| Age (years) of population at risk | 55–64 | | 65–74 | |
| Approximate mean age at risk | 60 | | 68 | |
| Age at starting to smoke | Under 15 | Over 25 | Under 15 | Over 25 |
| Approximate mean age at starting to smoke | 13 | 28 | 13 | 28 |
| Approximate duration of smoking (years) | 47 | 32 | 55 | 40 |
| Observed lung cancer death rate per 100,000 p.a. | 251 | 53 | 478 | 162 |
| Number of deaths from lung cancer | | | | |
| (a) Observed | 70 | 30 | 65 | 70 |
| (b) Expected* | 74·3 | 32·4 | 67·9 | 60·4 |

* Using the above approximate durations and assuming that lung cancer incidence rates are proportional to the fourth power of duration of smoking, and making the total expected numbers equal the total observed.

### MATERIALS AND METHODS

*Mice and details of treatment.*—The experiment was carried out in a closed animal unit under barrier conditions. Nine hundred and fifty female mice of a random-bred Swiss albino strain born in one part of the unit were, at the age of 5 ($\pm$ 1) weeks, transferred to a room in another part of the unit set aside for the experiment. At this time they were randomly allocated to groups 1, 2, 3 or 4, which consisted respectively of 140, 170, 220 and 420 mice. They were housed in macralon boxes, 10 per box, given autoclaved wood shavings as bedding, and provided *ad libitum* with water and an autoclaved vitamin fortified diet based on the 41B formula (supplied by Spillers Ltd). The normal lifespan of such mice in these conditions is 2–3 years.

At the age of 10 weeks, and at weekly intervals thereafter, the backs of the 140

mice of Group 1 were shaved from the neck to the root of the tail, using clippers lubricated with Liquid Paraffin, *B.P.C.* (which does not cause skin tumours when applied to the skin of mice). From the same age, 20 μg of benz(a)pyrene (BP) in 0·25 ml of acetone was applied twice each week by pipette to the shaved back. Previous experience with this treatment suggested that malignant skin tumours would be produced after 1–2 years of regular treatment. The BP was obtained from L. Light & Company, and the acetone (Analar grade) from Messrs Hopkins & Williams. At fortnightly intervals after the start of regular painting, " charting " occurred: the backs of the animals were examined and any visible and/or palpable lumps apparently arising from the epithelium were noted and measured. Three sets of callipers were available, set with gaps of 2 mm, 6 mm and 10 mm between blunted points, and at each fortnightly charting it was noted whether the diameters of lumps exceeded these calliper settings. When a lump which appeared to be arising from the epithelium exceeded 10 mm, the mouse was killed and sections 6 μm thick prepared from the lump and stained with haematoxylin and eosin for histological examination. The same procedure was adopted for the mice of Groups 2, 3 and 4, except that shaving and regular BP administration were started at 25 weeks of age in Group 2, at 40 weeks of age in Group 3, and at 55 weeks of age in Group 4.

All mice were examined at least once daily on 7 days per week for sickness or death. Sick mice were killed. No animal with a 10 mm diameter lump was lost because of cannibalism or advanced autolysis.

When animals died or were killed, lumps of any size in the subcutaneous tissues in the shaved area were taken for histological examination, in addition to 10 mm lumps apparently arising from the skin surface. Many of the subcutaneous lumps proved to be sarcomata and none were 10 mm epithelial tumours. However, 9 out of the total of 500 10 mm lumps which appeared to be arising from the epithelium were not, in fact, epithelial tumours: 8 were sarcomata and one was an ulcerating cyst associated with a malignant lymphoma.

*Statistical analysis.*—Because the observed incidence of new tumours during a particular fortnight may well be, for example, only 2/100, which is so small that it is unstable, we need some stable way of displaying the incidence rates in each of the 4 groups so that the overall pattern is not lost in random variation. The " cumulative incidence " (Peto, 1974) and the " life-table " (Pike and Roe, 1963) are equally useful for this purpose. Both use the fact that the sum of many small, individually unstable incidence rates is quite stable. The life-table gives the percentage of animals which would still be tumourless if the observed incidence rates affected a population of animals which did not die from other causes: although this is somewhat hypothetical, it does give us some feeling for what the life-table means, and so we have displayed our results by means of life-tables, calling the vertical axis " % mice without tumours ".

Although the main interest of the experiment was in the *qualitative* dependence of incidence on duration of treatment and on age given duration of treatment, a quantitative comparison between Group 1, which started regular treatment at 10 weeks of age, and Group 4, which started regular treatment at 55 weeks of age, was performed using methods appropriate for " non-incidental " tumours (Peto, 1974).

It made no material difference to the results of our analysis whether we compared " time to first 2 mm epithelial tumour ", " time to first 6 mm epithelial tumour " or " time to first 10 mm epithelial tumour " in the 4 groups. Of these analyses, " time to first 10 mm epithelial tumour " seemed the most biologically relevant, since a greater proportion of the 2 mm and 6 mm tumours were benign, and so this is the only one reported in detail.

Since tumours were " charted " only once every fortnight, if a tumour reached 10 mm between 2 chartings it would not be recorded as having done so until the second charting. Time is therefore divided into fortnights and we only know that 10 mm tumours detected at the end of a fortnight reached 10 mm at some time during that fortnight. The sizes of skin tumours in mice which died or were killed between chartings were measured and it was assumed that mice which died in the first half of the fortnight without 10 mm tumours had not been " at risk " for that fortnight while mice which died in the second half had been. In a few mice more than one skin tumour grew to a diameter of 10 mm or more during one fortnight. In these cases the animal was categorized using only the most malignant of its 10 mm skin tumours. Sections were obtained from all 10 mm tumours without exception, but sections from 10 of these tumours were lost. All 10 were, from the description of their appearance *in vivo*, epithelial tumours and have been assumed to be such in the presentation and analysis of the results.

### RESULTS

### *Histology of* 10 *mm epithelial tumours*

Almost all of these epithelial tumours were malignant: of the 481 from which sections reached the histologist, 427 (89%) showed infiltration of the panniculus muscle and in the opinion of the pathologist (FJCR) a further 42 (9%) were also malignant since they showed invasion of the dermal tissues, although not of the panniculus muscle. There would be no disagreement among pathologists about the malignant nature of the tumours which showed invasion of the panniculus, but the malignant status of some of the other tumours might be disputed. We therefore concluded only that over 90% of the 10 mm epithelial tumours were unequivocally malignant.

There was no difference between the 4 treatment groups in the proportions of 10 mm tumours which showed infiltration of muscle (Table II) and so our analysis would yield similar results even if we had restricted our interest still further to only those 10 mm epithelial tumours which were infiltrating the panniculus muscle.

### *Comparison of Hypotheses 1 and 2 for incidence rates of* 10 *mm epithelial tumours*

If benzpyrene acts on the first stage of the progression of normal cells towards malignancy, we would expect very different results under Hypotheses 1 and 2 if we plotted the life tables for the 4 treatment groups against age and then against duration of exposure. Figure 1 displays the expected pattern of results under Hypothesis 2 (in which incidence rate depends wholly on treatment duration and, given this, not at all on age) and under the most extreme version of Hypothesis 1 (in which incidence rate depends wholly on age and, given this, not at all on treatment duration); the actual results are set out in Fig. 2. The complete data from which Fig. 2 was derived are set out in the Appendix. It is clear that the extreme version of Hypothesis 1 is completely untenable: the increased rate of production of tumours after prolonged exposure to benzpyrene cannot plausibly be attributed to mice becoming more susceptible to benzpyrene as they grow older. Hypothesis 2 is extremely plausible, however: the response of mouse skin to benzpyrene can be explained very satisfactorily in terms of duration of exposure. There is in fact no indication at all of age having any substantial effect after duration of treatment is taken into account. The actual numbers of 10 mm epithelial tumours observed in the 4 groups are

TABLE II.—*Numbers of* 10 *mm Epithelial Tumours which were Found to have* (*a*) *Infiltrated the Panniculus Carnosus and* (*b*) *Not, by Treatment Group*

| Treatment group | No. of mice | Age (weeks) when regular application began | (a) Infiltrated | (b) Not | Percentage found to have infiltrated | Section lost |
|---|---|---|---|---|---|---|
| 1 | 140 | 10 | 93 | 12 | 89% | 5 |
| 2 | 170 | 25 | 115 | 13 | 90% | 0 |
| 3 | 220 | 40 | 99 | 16 | 86% | 4 |
| 4 | 420 | 55 | 120 | 13 | 90% | 1 |

FIG. 1.—Life tables depicting percentages of tumourless mice against (a) age, (b) duration of exposure to benzo(a)pyrene under Hypotheses 1 and 2.

displayed in Table III, together with the duration of exposure at which the life-table " per cent tumourless " reaches various values.

It is noteworthy that the life-table technique allows us to recognize that a crop of 134/420 (32%) in Group 4 and a crop of 110/140 (79%) in Group 1 actually represent very similar carcinogenic responses: the main tumour crop appeared after more than one year of treatment and these different absolute incidences arose merely because far more of the 420 animals of Group 4 which started exposure to BP at age 55 weeks died before they had a chance to develop a skin cancer. This similarity is emphasized by the fact that, using a logrank test (Peto and Pike, 1973), the difference between Groups 1 and 4 in time from starting BP to developing a tumour is not statistically significant ($P > 10\%$). The incidence in the older mice was, in fact, slightly but not significantly lower than in the younger mice with a similar duration of BP exposure.

*Comparison of Hypotheses 1 and 2 for tumour growth rates from* 2 *mm to* 10 *mm*

Another way in which ageing tissue might differ from younger tissue is in the rate at which established tumours grow. In laboratory animals exposed to carcinogens, it is commonly true that the tumours which arise early grow more rapidly than those that arise later. A possible explanation is that faster growth seen after a tumour has reached a detectable size is matched by faster growth from the time of induction to detectable size, *i.e.* rapid growth *caused* early appearance. Alternatively, the growth rates of tumours originating in ageing tissues might be essentially slower. The present experiment enables us to test these possible explanations, since we are able to compare the times taken to grow from 2 mm to 10 mm by tumours which reached

(a)

(b)

FIG. 2.—Actual forms of the life tables depicting percentages of tumourless mice against (a) age, (b) duration of exposure to benzo(a)pyrene in Groups 1–4.

TABLE III.—*Relationship of Crop of* 10 *mm Epithelial Tumours to Repeated Applications of Benzo(a)pyrene (BP) to the Skin*

| | Group 1 | Group 2 | Group 3 | Group 4 |
|---|---|---|---|---|
| Age (weeks) at start of regular BP | 10 | 25 | 40 | 55 |
| No. of animals allocated randomly (at age 5 weeks) to the four groups | 140 | 170 | 220 | 420 |
| No. which developed a 10 mm epithelial tumour | 110 | 128 | 119 | 134 |
| Life-table estimate of the duration (in weeks) of BP treatment by which, in the absence of other causes of death | | | | |
| (a) 1% of mice | 44 | 46 | 44 | 44 |
| (b) 33% of mice | 64 | 64 | 68 | 66 |
| (c) 67% of mice | 74 | 74 | 78 | 78 |
| would have got a 10 mm epithelial tumour. (The mice were examined fortnightly.) | | | | |

10 mm after similar durations of exposure to BP in mice whose ages differed by up to a year.

We find, as expected, that the mean time since a 10 mm epithelial tumour was 2 mm is shorter for the 10 mm tumours which arose earliest after the start of treatment; the regression of " time since 2 mm " on " treatment duration at 10 mm " in weeks is $10 + (\text{duration} - 70{\cdot}4) \times 0{\cdot}19$, the standard error of the regression coefficient 0·19 being $\pm 0{\cdot}02$ and the standard deviation of (actual–predicted) growth times being $\pm 5{\cdot}5$ weeks. However, it is not true that tumours which arise after similar durations of painting grow faster in younger animals; the simultaneous regression coefficients of " time since 2 mm " on " treatment duration at 10 mm " and " age at 10 mm " are $0{\cdot}190 \pm 0{\cdot}022$ and $-0{\cdot}006 \pm 0{\cdot}013$.

In summary, growth rates, like incidence rates, depend on duration of exposure to benzpyrene (the most rapidly growing tumours tending to be detected sooner) but not at all on age given duration of exposure.

*Test of the power-law increase of incidence rates with duration of exposure*

Since our aim in this experiment was to compare various explanations for the observed power-law increase with age of many human adult cancer rates (in which the incidence rate during adult life is approximately a power of age), we must check that our data exhibit, at least approximately, a power-law increase of cancer incidence rates with duration of exposure.

In the human situation, where the period of growth of a tumour may sometimes be only a small fraction of the duration of exposure, it may be reasonable under the local multistage hypothesis to expect a reasonably exact fit to this relationship. However, the situation in our experiment on mice is somewhat different.

We know that the time taken for tumours to grow from 2 mm diameter (about $10^6$ cells) to 10 mm diameter (about $10^8$ cells) is, on average, about 6 weeks for tumours arising after 50 weeks of BP treatment, about 10 weeks for tumours arising after 70 weeks of BP treatment and about 14 weeks for tumours arising after 90 weeks of treatment. In other words, a 100-fold increase in the numbers of cells is taking of order 2 or 3 months to complete. How long, on average, has the growth from a single malignant cell to a 10 mm tumour taken? This question cannot be answered directly but it seems unlikely to be less than 6 months and may well be considerably more, since the transition from a single cell to a 2 mm tumour requires three 100-fold increases and (since the one observable 100-fold increase takes 2 or 3 months) these three 100-fold in creases will presumably take at least 4 months to complete.

Because of this delay, we would not expect the age-specific incidence rate of the first tiny clones of malignant cells to be exactly reflected in the age-specific incidence rate of 10 mm epithelial tumours. Moreover, random variation in these delays will make the increase of the incidence rate of 10 mm tumours with exposure duration shallower than the increase of the incidence rate of first clones with exposure duration.

Despite these reservations, we have plotted, for each fortnightly charting, the percentage incidence of 10 mm epithelial tumours observed at that charting on a logarithmic scale against duration of BP exposure minus $6\frac{1}{2}$ months (the value which generated the straightest line), pooling all the data from Groups 1–4. The results of this are shown in Fig. 3, and it is obvious that over the 100-fold range from 0·25% per fortnight up to the massive incidence rate of 25% per fortnight observed after 90 weeks of regular BP administration, the points do approximately fit a straight line. Measurement of the slope of this line shows the best-fitting whole number is three. This means that the cancer incidence rates do

FIG. 3.—Incidence rates (as percentages) of 10 mm epithelial tumours at successive fortnightly chartings against duration of BP application, on a log/log scale from 28 weeks. These rates, calculated from the pooled data of all 4 treatment groups, are statistically independent of each other and 90% confidence intervals are indicated.

increase approximately as the third power of (duration — 28 weeks). Our data may therefore be relevant to the power-law increase of human cancer incidence rates with age. The only anomalies are at weeks 50 and 70, and these are not serious in view of the large number of independent rates which are being examined. A single mouse in Group 4 developed a 10 mm infiltrating epithelial tumour at age 79 weeks in treatment week 24: this was perhaps spontaneous, as such tumours do arise in about 0·5% of untreated control mice of this strain, but apart from this no 10 mm epithelial tumours arose before the 40th week of treatment. After the 90th week of treatment, only 15 mice remained alive, 12 of which already had epithelial tumours of less than 10 mm diameter, and the calculation of meaningful incidence rates became impossible.

*Body weight*

Could differences in body weight and growth rates between groups invalidate the conclusions that we have so far reached?

At the age of 10 weeks the mean weight of the 950 mice in the experiment was 28·8 g. Group 1, which received BP from 10 weeks of age, grew more slowly than the other groups. The respective mean weights in grams of the survivors in the four groups at 55 and 75 weeks of age were as follows:

| Age (weeks) | Group 1 | 2 | 3 | 4 |
|---|---|---|---|---|
| 55 | 41·1 (125 mice) | 42·5 (156 mice) | 43·2 (200 mice) | 45·3 (401 mice) |
| 75 | 42·8 (80 mice) | 44·0 (141 mice) | 46·8 (194 mice) | 47·9 (377 mice) |

The application of BP to the skin of Groups 1–3 between the ages of 10 weeks and 55 weeks was associated with a slight reduction in growth. Weight gain in Groups 2 and 3, in which BP treatment was started at 25 and 40 weeks respectively, was intermediate between that in Group 1 and that in Group 4. We do not know whether these differences were associated with differences in the amounts of food consumed. It is concluded that although differences in growth might have slightly influenced the tumour crops detected, the effect is unlikely to have been enough to affect our qualitative conclusions.

*Sarcomata*

A total of 44 of the 950 mice in the experiment developed sarcomata arising in the subcutaneous tissues (1 mouse in Group 1, 3 in Group 2, 10 in Group 3 and 30 in Group 4, between weeks 69 and 121 of age—see Appendix). The sarcoma incidence rate was found to depend on age but, given age, not on the duration of BP treatment ($\chi_3{}^2$ comparing age-specific sarcoma incidence rates in the 4 groups = 0·4, indicating no heterogeneity). This result could be explained theoretically in one of 2 ways: either BP has no effect on sarcoma incidence at all or BP affects only the final stage of the carcinogenic process, while the earlier stages are brought about by influences other than BP. From our experimental results alone, it is not possible to discriminate between these 2 possibilities as there was no untreated control group. In previous skin-painting experiments (*e.g.* Roe *et al.*, 1970), female Swiss mice exposed only to acetone experienced age-specific incidence rates of the same order as in the present experiment. It seems likely therefore that BP has no, or at most a very small, effect on sarcoma incidence. As with human sarcomata, the incidence rate of sarcomata in our experiment increased less steeply with time than did the incidence rates of epithelial tumours.

### DISCUSSION

We have applied a carcinogen to the skin of mice up to the extremes of old age (*i.e.* 2–3 years) and have observed that the skin cancer incidence rates depend only on duration of carcinogenic insult and, given this duration, not at all on the age of the animals when regular exposure to the insult began (which was 10, 25, 40 or 55 weeks). These results can be completely explained by the local multistage model with no intrinsic effect of age. Although it is possible that an intrinsic effect of ageing was obscured by opposing age-related changes in metabolic activation, deactivation or clearance rates, hair growth cycles and so on which are peculiar to our experiment and which would not necessarily be relevant to other carcinogens, to other species or to target tissues other than the skin, there is no positive reason to suppose that any such biases have occurred.

*Comparison with other experimental findings*

A result which apparently contradicts our findings has been reported by Ebbesen (1973, 1974), who studied DMBA induced carcinomata in mouse skin of different ages transplanted on to syngeneic hosts of the same age. Although the experimental methods were not reported in sufficient detail to allow proper evaluation of the results, it appears that the older the skin at the time of transplant, the more susceptible it was to subsequent tumour induction. Like us, Ebbesen compared the incidence of skin carcinomata in skin aged 1–2 years with the incidence in skin aged 2–3 years, but his carcinogenic stimulus was weaker than ours (2 isolated DMBA applications resulting in only 10% or so of the animals developing carcinomata) and it may be that our stronger stimulus swamped the spontaneous mutations in the stem cells of the skin whereas his weaker stimulus did not. In any case, his results can be explained naturally under Hypothesis 2 while our results cannot under Hypothesis 1.

However, this explanation raises rather a delicate point. In our experiment, as in Ebbesen's experiment, carcinogens are applied to tissues of different ages to discover whether, apart from the accumulation with time of heritable changes in particular cells, age has any *other*

relevance to carcinogensis. If no difference is observed, as in our experiment, that is evidence that age has no other relevance and that the spontaneous rates of each stage were swamped by the carcinogen induced rates of those stages. If, however, the older animals are more vulnerable, as in Ebbesen's experiment, this can be interpreted as evidence that the spontaneous accumulation of certain stages with time is sufficient not to be swamped by the carcinogen and that although no other effect of age is relevant, this accumulation is sufficient to generate an appreciable difference in vulnerability. Can the hypothesis of " no *other* relevance of age " (Hypothesis 2) ever be contradicted by any conceivable result? It can, but not by applying carcinogens to tissues of different ages, as we have done; our experiment can only contradict Hypothesis 1. A critical experiment for Hypothesis 2 is to apply carcinogens to tissues of the same age transplanted into hosts of different ages, but as far as we know no such experiment has ever been reported.

Another result like Ebbesen's is that of Berry and Wagner (1975), who have examined the induction of rat mesotheliomata by a single intrapleural instillation of asbestos at either 2 or 10 months of age. If it is assumed that the asbestos is not eliminated, this constitutes a continuous carcinogenic stimulus, starting at the time of the instillation and continuing until death and it appears, on preliminary inspection of their data, that the group treated later in life developed mesotheliomata more rapidly after the instillation than did the other group. Again, however, this can be explained under Hypothesis 2 by assuming that some of the stages occurred spontaneously to an appreciable extent between months 2 and 10. The experiment of Van Duuren *et al.* (1975) on 561 mice may represent a third such result, but unfortunately failure to use life-tables in describing their findings makes it impossible to discover whether any age-related differences in tumour inducibility existed.

Finally, Meranze *et al.* (1969) may have found one genuine age-related change in tumour inducibility. 15 mg of intragastric DMBA results in more mammary tumours in female rats if given at about 6 weeks of age than if given at about 26 weeks of age, even if differences in subsequent duration of exposure are properly allowed for. This may be due to age-related hormonal changes which selectively affect mammary tissue, as in adult human females. However, DMBA in such enormous doses has many other systemic effects, particularly on hormonal status, and it may be these rather than any more direct carcinogenic effects which are responsible. Moreover, the dose in mg *per kg body weight* was, of course, less than half as big in their older animals, and it is also possible that all Meranze *et al.* observed was an age related change in the effective dose to the target organ.*

### *Conclusions*

Notwithstanding these doubts, our results certainly show that a power law increase of incidence with duration of exposure can arise in the absence of any intrinsic effects of age such as, for example, decreasing efficiency of immunological surveillance. (Some independent evidence against the influence, age dependent or otherwise, of immunological surveillance on cancer incidence already exists, in that Rygaard and Povlsen (1974) have shown that mice which were genetically incapable of making their full complement of T lymphocytes nevertheless suffered no appreciable excess of spontaneous tumours.) By default of other explanations, the increase of those cancer incidence rates which increase approximately as a power of age appears to occur merely because as time passes exposure to car-

* The other difficulty that can arise in experiments on the effects of age on the induction of internal neoplasms, as opposed to skin cancers, is that the greater intercurrent death rates in old animals may cause more of their internal tumours to be discovered early.

cinogens and to mistakes in gene replication during routine mitosis continues, and heritable changes thus accumulate. It could still be postulated that the natural kinetic rate-constants of the stages strongly affected by our carcinogen do increase with age, but there seems no reason to do so. Our results thus lend some support to the local multistage hypothesis according to which the kinetic rate-constants of each stage are (given which other stages have occurred) largely independent of age and depend mainly on the presence and amounts of various carcinogens and, perhaps, on the rate of stem cell division in the target tissue.

*Human exposure at different ages*

Under almost any hypothesis, lifelong regular exposure of humans to a carcinogenic insult will be more dangerous the younger exposure starts. There is, however, disagreement as to whether brief exposure is more dangerous for the young or for the old. Our results suggest that although the immediate risk to old people may be greater, the total subsequent effect of brief exposure to a carcinogenic insult will eventually be worse in people exposed when young. (This might not be true for a brief insult which merely " promotes " and cannot " initiate ", but conversely an insult which merely initiates and cannot promote would be enormously more dangerous for people who were exposed to it when young.)

*An unresolved difficulty*

We have shown that the lapse of an extra year of age before starting treatment (more than one-third of the life span) does not increase the susceptibility of mouse skin to benzpyrene carcinogenesis, nor does it increase the rate of growth of such tumours once they are established. Previous work (Lee and O'Neill, 1971) has shown that in this system the age specific incidence rate is proportional to the *square* of the dose rate of benzpyrene and it can be shown that under a simple hypothesis such as that of Armitage and Doll (1961) this implies that benzpyrene strongly affects at most *two* distinct stages. (This conclusion is reinforced by unpublished studies now being analysed by one of us (PNL) on the pattern of tumour incidence following discontinuation of benzpyrene treatment in mice.) Taken together, these observations suggest either that there are only 2 stages, in which case it is very difficult to find models which lead to a third power law increase with age, or that, if there are more than 2 stages, benzpyrene acts on the first stage (and on one later stage) and that other stages cannot take place until this first benzpyrene related stage is complete. Although this sounds rather implausible (why should certain mutation rates be increased by benzpyrene while others are not?), the next paragraph shows that it is not impossible.

A diploid cell has 2 loci that code for each enzyme, one on each of a pair of homologous chromosomes. If inactivation of such an enzyme is required during carcinogenesis, this must occur in 2 stages. In the first stage, the carcinogen effects a destructive mutation in one of the loci, converting the cell from AA to $A\bar{A}$ ($\bar{A}$ being inactive). The second stage might then take place not by a second destructive mutation but rather by " mitotic recombination ", a rare error whereby mitosis results in AA and $\bar{A}\bar{A}$ instead of 2 $A\bar{A}$ daughters. This provides a natural mechanism whereby no exogenous carcinogen is required to bring about some of the stages. However, although such a sequence of events could explain the third-power dependence of cancer incidence on duration of exposure observed when benzpyrene is applied to mouse skin, it is difficult to see how it alone could lead to the fifth- and sixth-power dependence on duration sometimes observed in man, especially since, in the most extensively studied human situation (the association between cigarette smoking and lung cancer), the cancer incidence rate appears to increase only as a single power of dose. The fact that chronic exposure to a carcinogen leads to an incidence rate of cancer which depends far more strongly on duration of exposure than on dose rate is one of the most important unexplained features of the aetiology of cancer in adults.

*A possible non-causal link between ageing and cancer incidence*

We have concluded that during normal life, random heritable changes may occur by accident in particular stem cells and that when certain such changes all happen in a single stem cell the result may be proliferation into a recognizable cancer. However, of all the heritable changes that might occur in a stem cell, the majority will presumably be irrelevant to carcinogenesis and so, ignoring lethal changes which would eliminate a cell line, in old age several stem cells must have suffered many non-lethal heritable changes by the same general mechanisms that are involved in carcinogenesis. Burnet (1974) has suggested that this accumulation of somatic changes is the fundamental process of ageing, and that the rate at which they occur is subject to evolutionary control (perhaps by inter-species differences in the efficiency of the DNA repair enzymes) and largely determines the typical lifespan of each species. These evolved controls on the rates at which random heritable changes arise in the stem cells of an organism would presumably affect not only the timespan of ageing, but also that of carcinogenesis. If this is so, then the rate of generation of somatic mutations determines both the rate of ageing and the age specific incidence rate of cancer. This postulated common cause for ageing rates and for cancer incidence rates might be the reason why most species suffer some cancer of old age, whether old age occurs at 80 weeks or 80 years, even though ageing itself does not affect oncogenesis.

This work was supported by grants from the Cancer Research Campaign, the Medical Research Council, and the Tobacco Research Council to the Chester Beatty Research Institute, for which we are extremely grateful. We have received helpful criticism of this work from Sir Richard Doll, Laszlo Lajtha, Nick Wald, Gerald Draper, Bruce Armstrong, Alice Whittemore, Howard Cuckle, John Cairns, John Mathews and Sir F. Macfarlane Burnet.

### REFERENCES

ARMITAGE, P. & DOLL, R. (1961) Stochastic Models for Carcinogenesis. In *Proc. Fourth Berkeley Symp. Mathematical Statistics and Probability.* IV. Biology and Problems of Health, p. 19.

BERENBLUM, I. & SHUBIK, P. (1947) The Role of Croton Oil Application, Associated with a Single Painting of a Carcinogen, in Tumour Induction of the Mouse's Skin. *Br. J. Cancer*, **1**, 379.

BERRY, G. & WAGNER, J. C. (1975) Effect of Age at Inoculation of Asbestos on Occurrence of Mesotheliomas in Rats. Revision in preparation for *Int. J. Cancer*.

BURNET, F. M. (1970) *Immunological Surveillance.* Oxford: Pergamon Press. Chap. 8.

BURNET, F. M. (1974) Intrinsic Mutagenesis, an Interpretation of the Pathogenesis of Xeroderma Pigmentosum. *Lancet*, ii, 495.

CAIRNS, J. (1975) Mutation, Selection and the Natural History of Cancer. *Nature, Lond.*, **255**, 197.

DILMAN, V. M. (1971) Age-associated Elevation of Hypothalamic Threshold to Feedback Control and its Role in Development, Ageing and Disease. *Lancet*, i, 1211.

DOLL, R. (1971*a*) Cancer and Ageing: the Epidemiologic Evidence. In *Oncology*, 1970, **5**, 1. Chicago: Yearbook Medical Press.

DOLL, R. (1971*b*) The Age Distribution of Cancer (with Discussion). *J. R. stat. Soc.* A, **134**, 133.

DOLL, R., MUIR, C. & WATERHOUSE, J. (Eds) (1970) *Cancer Incidence in Five Continents.* UICC.

EBBESEN, P. (1973) Papilloma Induction in Different Aged Skin Grafts to Young Recipients. *Nature, Lond.*, **241**, 280.

EBBESEN, P. (1974) Ageing Increases Susceptibility of Mouse Skin to DMBA Carcinogenesis Independent of General Immune Status. *Science, N.Y.*, **183**, 217.

FIALKOW, P. J. (1974) The Origin and Development of Human Tumours Studied with Cell Markers. *New Engl. J. Med.*, **291**, 26.

KAHN, H. A. (1966) The Dorn Study of Smoking Mortality among US Veterans: Report on Eight and one-half Years of Observation. In *Epidemiological Study of Cancer and Other Chronic Diseases. Natn. Cancer Inst. Monog.*, **19**, 1.

LEE, P. N. & O'NEILL, J. A. (1971) The Effect both of Time and Dose Applied on Tumour Incidence Rates in Benzpyrene Skin Painting Experiments. *Br. J. Cancer*, **25**, 759.

LEE, P. N. & PETO, R. (1970) The Effect of Age of Mice on the Incidence of Skin Cancer. *Br. J. Cancer*, **24**, 849.

MERANZE, D. R., GRUENSTEIN, M. & SHIMKIN, M. B. (1969) Effect of Age and Sex on the Development of Neoplasms in Wistar Rats receiving a Single Intragastric Instillation of 7,12-dimethylbenz(a)-anthracene. *Int. J. Cancer*, **4**, 480.
PETO, R. (1974) Guidelines on the Analysis of Tumour Rates and Death Rates in Experimental Animals. *Br. J. Cancer*, **29**, 101.
PETO, R. & LEE, P. N. (1973) Weibull Distributions for Continuous-Carcinogenesis Experiments. *Biometrics*, **29**, 457.
PETO, R. & PIKE, M. C. (1973) Conservatism of the Approximation $\Sigma(O-E)^2/E$ in the Logrank Test for Survival Data or Tumor Incidence Data. *Biometrics*, **29**, 579.
PIKE, M. C. & ROE, F. J. C. (1963) An Actuarial Method of Analysis of an Experiment in Two-stage Carcinogenesis. *Br. J. Cancer*, **17**, 605.
ROE, F. J. C. (1959) The Effect of Applying Croton Oil Before a Single Application of 9,10-dimethyl-1,2-benzanthracene (DMBA). *Br. J. Cancer*, **13**, 87.
ROE, F. J. C., CLACK, J. C., BISHOP, D. & PETO, R. (1970) Comparative Carcinogenicity for Mouse-skin of Smoke Condensate Prepared from Cigarettes made from the Same Tobacco Cured by Two Processes. *Br. J. Cancer*, **24**, 107.
RYGAARD, J. & POVLSEN, C. O. (1974) The Mouse Mutant *nude* Does Not Develop Spontaneous Tumours: an Argument Against Immunological Surveillance. *Acta path. microbiol. scand.*, Sect. B, **82**, 99.
VAN DUUREN, B. L., SIVAK, A., KATZ, C., SEIDMAN, I. & MELCHIONNE, S. (1975) The Effect of Ageing and Interval between Primary and Secondary Treatment in Two-stage Carcinogenesis on Mouse Skin. *Cancer Res.*, **35**, 502.

### APPENDIX

Complete data on the incidence of 10 mm epithelial tumours in the 4 treatment groups, giving numbers of 10 mm epithelial tumours and of animals charted for each fortnightly charting at which some 10 mm tumours were found. (The numbers of first sarcomata are given in brackets at the times when they were first visible, which usually preceded death by about 5 weeks.)

| Weeks of benzpyrene | Group 1 (BP from age 10 weeks) | Group 2 (BP from age 25 weeks) | Group 3 (BP from age 40 weeks) | Group 4 (BP from age 55 weeks) |
|---|---|---|---|---|
| 24 | 0/135 | 0/158 | 0/196 | 1/369 |
| 40 | 0/130 | 0/154 | 1/192 (2[a]) | 1/311 (8[b]) |
| 42 | 1/128 | 1/154 | 0/190 | 1/303 |
| 44 | 1/126 | 0/150 | 1/187 | 3/296 (1) |
| 46 | 2/125 | 2/149 | 0/182 | 2/286 (1) |
| 48 | 2/123 | 5/146 | 1/181 | 2/275 (2) |
| 50 | 2/121 | 0/141 | 0/176 | 3/265 (4) |
| 52 | 6/119 | 4/141 | 2/172 | 1/255 |
| 54 | 2/113 | 3/137 | 1/116 | 6/239 (1) |
| 56 | 3/111 | 4/130 | 4/162 (1) | 9/222 (1) |
| 58 | 10/108 | 4/126 | 4/154 (3) | 10/203 (4) |
| 60 | 8/98 | 9/120 | 7/149 | 12/185 |
| 62 | 5/89 | 7/109 | 5/137 (1) | 14/160 (3) |
| 64 | 2/82 | 9/100 (1) | 12/129 | 9/133 (2) |
| 66 | 10/80 | 6/88 | 8/110 (1) | 9/110 (3) |
| 68 | 7/70 | 10/81 | 7/97 | 11/98 |
| 70 | 9/62 | 17/69 (1) | 9/87 (2) | 10/77 |
| 72 | 8/52 | 6/51 (1) | 7/74 | 7/63 |
| 74 | 7/43 | 8/45 | 8/65 | 6/55 |
| 76 | 1/35 | 7/37 | 7/51 | 1/42 |
| 78 | 4/33 | 6/30 | 9/42 | 6/34 |
| 80 | 4/28 (1) | 3/23 | 1/33 | 7/23 |
| 82 | 3/23 | 3/20 | 6/32 | 1/13 |
| 84 | 4/15 | 4/16 | 6/25 | 0/12 |
| 86 | 4/10 | 4/12 | 2/18 | 0/11 |
| 88 | 1/6 | 3/8 | 4/15 | 2/10* |
| 90 | 2/5 | 3/4 | 0/11 | |
| 92 | 0/3 | 0/1 | 0/9 | |
| 94 | 0/3 | 0/1 | 5/9 | |
| 96 | 0/3 | 0/1 | 2/3 | |
| 98 | 0/2 | 0/1 | 0/1 | |
| 100 | 2/2 | 0/1 | 0/1 | |
| 102 | 0/0 | 0/1 | 0/1 | |
| 104 | 0/0 | 0/1† | 0/1* | |

* End of experiment.
† Lived without 10 mm tumour to end of experiment at treatment week 118.
[a] Weeks 34, 36 of benzpyrene.
[b] Weeks 14, 14, 18, 22, 36, 36, 38, 38.

*Notes added in proof*

(1) At any given age, there were no systematic differences between the four groups in their death rates from causes other than 10 mm epithelial tumours. (The logrank chi-square for trend between the four groups in mortality from such causes was 0·00, showing no trend whatever.) This suggests that animals only died of treatment *via* the induction of epithelial tumours, and supports our belief that there was no tendency to die of epithelial tumours before they reached 10 mm. (One would expect, in an inbred strain, little heterogeneity in susceptibility to ageing or to carcinogenesis, and hence no appreciable within-strain correlation between these two susceptibilities. This expectation is also supported by the above null trend.)

(2) Had a Weibull distribution been fitted to these data by maximum likelihood, as suggested by Peto and Lee (1973), the absolute values of the parameters $k$ and $w$ would have been unduly influenced by the single tumourless survivor in group 2, and still more by the early tumour in group 4. This emphasizes the importance of comparing Weibull $b$-parameters by Cox's method whenever possible (*ibid*., p. 468).

(3) It has been suggested that heritable changes affecting DNA repair enzymes might accumulate with age, and that when sufficient such changes have accumulated, successful cell division is unlikely. This postulated process (or a modified version of it in which the DNA repair enzymes are so damaged that further damage to the DNA coding for these enzymes then occurs rapidly) is referred to as " error catastrophe ". It has been suggested that error catastrophe is responsible for tissue ageing, and that it is responsible for the increased cancer incidence rates among old individuals. As we have described error catastrophe, changes in one cell only affect its own descendants and error catastrophe is merely a particular example of our " local multistage model " in which the early stage(s) affect the repair enzymes. If, implausibly, some form of change which would affect DNA repair in all the cells in a tissue similarly were postulated, then older tissues would be essentially more vulnerable to carcinogens in a manner which has been contradicted by our experiment.